# Do beaver dams reduce habitat connectivity and salmon productivity in expansive river floodplains?

**DOI:** 10.7717/peerj.2403

**Published:** 2016-09-01

**Authors:** Rachel L. Malison, Kirill V. Kuzishchin, Jack A. Stanford

**Affiliations:** 1 Flathead Lake Biological Station, University of Montana, Polson, MT, United States; 2 Ichthyology Department, Moscow State University, Moscow, Russian Federation; 3 Current affiliation: Norwegian Institute for Nature Research, Trondheim, Norway

**Keywords:** Alaska, Alluvial river floodplains, Kamchatka, North American beaver, *Castor canadensis*, Pacific salmon, Salmon ecology, Salmon production

## Abstract

Beaver have expanded in their native habitats throughout the northern hemisphere in recent decades following reductions in trapping and reintroduction efforts. Beaver have the potential to strongly influence salmon populations in the side channels of large alluvial rivers by building dams that create pond complexes. Pond habitat may improve salmon productivity or the presence of dams may reduce productivity if dams limit habitat connectivity and inhibit fish passage. Our intent in this paper is to contrast the habitat use and production of juvenile salmon on expansive floodplains of two geomorphically similar salmon rivers: the Kol River in Kamchatka, Russia (no beavers) and the Kwethluk River in Alaska (abundant beavers), and thereby provide a case study on how beavers may influence salmonids in large floodplain rivers. We examined important rearing habitats in each floodplain, including springbrooks, beaver ponds, beaver-influenced springbrooks, and shallow shorelines of the river channel. Juvenile coho salmon dominated fish assemblages in all habitats in both rivers but other species were present. Salmon density was similar in all habitat types in the Kol, but in the Kwethluk coho and Chinook densities were 3–12× lower in mid- and late-successional beaver ponds than in springbrook and main channel habitats. In the Kol, coho condition (length: weight ratios) was similar among habitats, but Chinook condition was highest in orthofluvial springbrooks. In the Kwethluk, Chinook condition was similar among habitats, but coho condition was lowest in main channel versus other habitats (0.89 vs. 0.99–1.10). Densities of juvenile salmon were extremely low in beaver ponds located behind numerous dams in the orthofluvial zone of the Kwethluk River floodplain, whereas juvenile salmon were abundant in habitats throughout the entire floodplain in the Kol River. If beavers were not present on the Kwethluk, floodplain habitats would be fully interconnected and theoretically could produce 2× the biomass (between June–August, 1,174 vs. 667 kg) and rear 3× the number of salmon (370,000 vs. 140,000) compared to the existing condition with dams present. The highly productive Kol river produces an order of magnitude more salmon biomass and rears 40× the individuals compared to the Kwethluk. If beavers were introduced to the Kol River, we estimate that off-channel habitats would produce half as much biomass (2,705 vs. 5,404 kg) and 3× fewer individuals (1,482,346 vs. 4,856,956) owing to conversion of inter-connected, productive springbrooks into inaccessible pond complexes. We concluded that beaver dams may limit the total amount of floodplain habitat available for salmon rearing in the Kwethluk river and that the introduction of beavers to the Kol river could be detrimental to salmon populations. The introduction of beavers to other large alluvial rivers like those found in Kamchatka could have negative consequences for salmon production.

## Introduction

Beaver (*Castor canadensis* and *C. fiber*) have expanded in their native ranges in recent decades through widespread reintroductions and natural spread following previous suppression by trapping (Halley, Rosell & Saveljev, 2012; Whitfield et al., 2015). Beaver are native in North America (*C. canadensis*) and Europe and Asia (*C. fiber*), and were introduced to Tierra del Fuego, Argentina in the 1960s where they spread rapidly. Populations in North America, Europe and Asia were heavily depleted by fur trapping, to the point that many populations were lost and in some cases, beaver were in danger of becoming extinct. However, following the relaxation of trapping and increase in reintroduction efforts, populations have grown rapidly, rebounding towards historic population levels. For example, Eurasian beavers recovered from a total of about 1,200 animals at the beginning of the 20th century to more than a million individuals by 2010 (Halley, Rosell & Saveljev, 2012). The reintroduction of beavers is currently being used in the western United States as a restoration technique for improving salmon habitat in low order streams (Pollock et al., 2012; Petro, Taylor & Sanchez, 2015), and the addition of dams on highly incised low order streams has been shown to have strong positive effects on stream habitat (Bouwes et al., 2016).

Beavers can strongly modify the physical and biological components of stream ecosystems by impounding water (Naiman, Johnston & Kelley, 1988), with consequences for stream fishes. By cutting vegetation and building dams beavers can alter the hydrology of riparian zones, resulting in increased biocomplexity of low order streams (Naiman & Rogers, 1997; Gurnell, 1998; Wright, Jones & Flecker, 2002; Rosell et al., 2005). The influence of beavers on stream fishes has been primarily studied in low order streams, where the impacts of beavers are generally positive (Kemp et al., 2012). The presence of beaver ponds may result in improved habitat quality and increases in invertebrate food resources (Hanson & Campbell, 1963; Keast & Fox, 1990), increased density and sizes of fish, including salmon (Gard, 1961; Hanson & Campbell, 1963; Bryant, 1983; Murphy et al., 1989; Leidholt Bruner, Hibbs & McComb, 1992; Schlosser, 1995; Bouwes et al., 2016), higher survival rates (Bustard & Narver, 1975; Quinn & Peterson, 1996) and faster growth rates for juvenile salmon (Bustard & Narver, 1975; Swales & Levings, 1989; Malison, Eby & Stanford, 2015). Other studies have also documented increased production in ponded habitat (Nickelson et al., 1992; Layman & Smith, 2001; Pollock et al., 2004; Bouwes et al., 2016). On the other hand, Snodgrass & Meffe (1998) found that that some beaver ponds (e.g., old active ponds) had decreased dissolved oxygen concentrations and lower fish species richness. However, despite reduced richness in these habitats, the presence of beaver ponds tended to increase fish diversity overall in headwater streams. In fact, the presence of beavers increases habitat complexity spatially and temporally, resulting in productive and diverse fish assemblages in low order streams in north-temperature areas (Schlosser & Kallemeyn, 2000). Though the benefits of beaver reintroductions likely outweigh the costs in low order stream systems (Kemp et al., 2012), it is not clear how expanding or introduced beaver populations may influence salmonid populations on large alluvial rivers with complex floodplains.

The expansive floodplains of large alluvial rivers provide rearing habitat important for the production of wild salmonids. Currently, forty-percent of all wild Pacific salmon are produced in the rivers of Kamchatka, Russia, with most of the rest coming from the large river and lake systems of Alaska and British Columbia (Augerot, 2005). Many of these rivers are large expansive floodplain systems (Whited et al., 2012). These dynamic floodplain environments are maintained by processes of cut and fill alluviation, channel avulsion, riparian plant succession, ground- and surface water exchanges, and erosion and deposition of wood that interact to create a complex shifting habitat mosaic (Stanford, Lorang & Hauer, 2005). Springbrooks are a dominant habitat type in expansive floodplains (Whited et al., 2013) and are known to be primary rearing areas for all species of Pacific salmon and trout *Oncorhynchus* spp., and char *Salvelinus* spp. (Eberle & Stanford, 2010; Armstrong & Schindler, 2013). Springbrooks have warm winter and cool summer temperatures from upwelling floodplain aquifer water and also have high ecological connectivity with riparian food webs; therefore enhanced growth and survival of juvenile salmon have been documented in these habitats (Sommer et al., 2001; Jeffres, Opperman & Moyle, 2008; Bellmore et al., 2013).

In the large alluvial rivers of Kamchatka salmon have evolved without beavers. In these floodplain systems springbrooks are present throughout the floodplain, both in the parafluvial (frequently scoured zone near the main channel) and orthofluvial (depositional zone farther from the main river) portions of the floodplain. These shallow springbrooks are connected to the main river channel and have high ecological connectivity with riparian food webs, providing ideal habitat for rearing juvenile salmon throughout the floodplain. However, floodplain springbrooks also provide ideal habitats for beaver damming due to their manageable flow levels and upwelling groundwater.

In contrast to Kamchatka, in North American rivers, beavers typically dam floodplain springbrooks creating extensive pond complexes that dominate the floodplain habitat mosaic (sensu Stanford, Lorang & Hauer, 2005). These beaver complexes can even be the predominant off-channel habitat in floodplain rivers. In the Kwethluk River, western Alaska, Malison et al. (2014) found that 80% of off channel habitat was comprised of beaver ponds, predominantly in the orthofluvial zone (depositional area farther from the main river). Furthermore, very few or no juvenile salmon were found in mid- and late-successional beaver ponds located in the orthofluvial zone, suggesting that those beaver ponds were either unproductive (e.g., de-oxygenated owing to organic matter decomposition) or inaccessible. In contrast, Malison, Eby & Stanford (2015) found that juvenile salmon rearing in early-successional beaver ponds within the parafluvial zone (near the main channel) of the Kwethluk River had increased growth rates and that these habitats contributed to increased salmon production for the parafluvial zone of the river. On another large alluvial river floodplain on the Taku River in Alaska, juvenile salmon were also found to be abundant in parafluvial beaver ponds (Murphy et al., 1989). Overall, the scale at which the impact of beavers on salmon production is measured becomes important. It is difficult to determine exactly how the loss of orthofluvial springbrooks (replaced by unproductive beaver ponds) may influence salmon production in large alluvial floodplains. For example, if beavers were removed would juvenile salmon be found in similar densities within orthofluvial springbrooks as they are in parafluvial springbrooks close to the main channel? Or are orthofluvial springbrooks generally a less important habitat because they are located farther from the main channel? The influence of beavers on salmonids in dynamic large alluvial rivers could be different than in low order streams and it is not clear how beavers may impact salmon in floodplain habitats of productive salmon rivers of the Pacific Rim.

Beaver populations have expanded in Alaska since the collapse of the fur trade and it is possible that the conversion of floodplain springbrooks into ponds by beavers may limit salmon production. In an experimental setting, measuring total salmon production in one or more beaver-modified rivers prior to and following complete removal of beavers would be ideal; but this approach is simply not feasible owing to the massive scale (hundreds of km^2^) of beaver activity in these floodplain settings (Malison et al., 2014). Alternatively, the absence of beavers in salmon rivers of the Kamchatka Peninsula (Russian Federation), where beavers have never existed and have not been introduced (Halley, Rosell & Saveljev, 2012) offers an interesting comparison to shed light on the issue, as well as provide insight regarding potential consequences of introducing beavers to these rivers.

Our intent in this paper is to contrast the habitat use by juvenile salmon on expansive floodplains of two geomorphically similar salmon rivers: the Kol River in Kamchatka, Russia (no beavers) and the Kwethluk River in Alaska (abundant beavers), and thereby provide a conservation perspective on the likely influences that beavers have on large floodplain rivers. We use this comparison to infer how beavers may impact salmon production in two rivers with similar geomorphology but with very different levels of salmonid productivity. The differing salmonid productivity levels are influenced by varying harvest levels, species composition of the ichthyofaunal and supporting food webs, and the import of marine derived nutrients, with the primary difference being the abundant pink salmon runs in the Kol River that can be up to 35 times larger than the total return of all five salmon species on the Kwethluk River on even years. Understanding how juvenile salmon use springbrooks within the parafluvial zone and orthofluvial zone of a beaver-free floodplain may allow us to infer how habitat use and salmon production might be different in the Kwethluk River if beavers were not present (or were present in much lower numbers). Furthermore, measuring how salmon use springbrooks and beaver ponds within the parafluvial and orthofluvial zones of the Kwethluk River will allow us to infer how productivity might differ in the Kol River if beavers were introduced. We predicted that (1) the productivity of coho (*O. kisutch*) and Chinook (*O. tshawytscha*) would be greater in the Kwethluk if beavers did not block large portions of the floodplain from salmon use and (2) that productivity would be much lower in the Kol if beavers were introduced. We measured fish species composition, densities, condition, and growth of juvenile coho and Chinook salmon in different types of rearing habitats (main channel shallow shorelines, tributaries, parafluvial and orthofluvial springbrooks, and early-, mid- and late-successional beaver ponds) within the parafluvial and orthofluvial zones of the two rivers. We used these data to assess how beaver dams may influence the production of juvenile salmon at the floodplain scale for each river. We focused the analysis on juvenile coho and Chinook because they rear in floodplain habitats for longer periods than other salmon species and therefore may be most strongly influenced by beavers.

**Figure 1:**
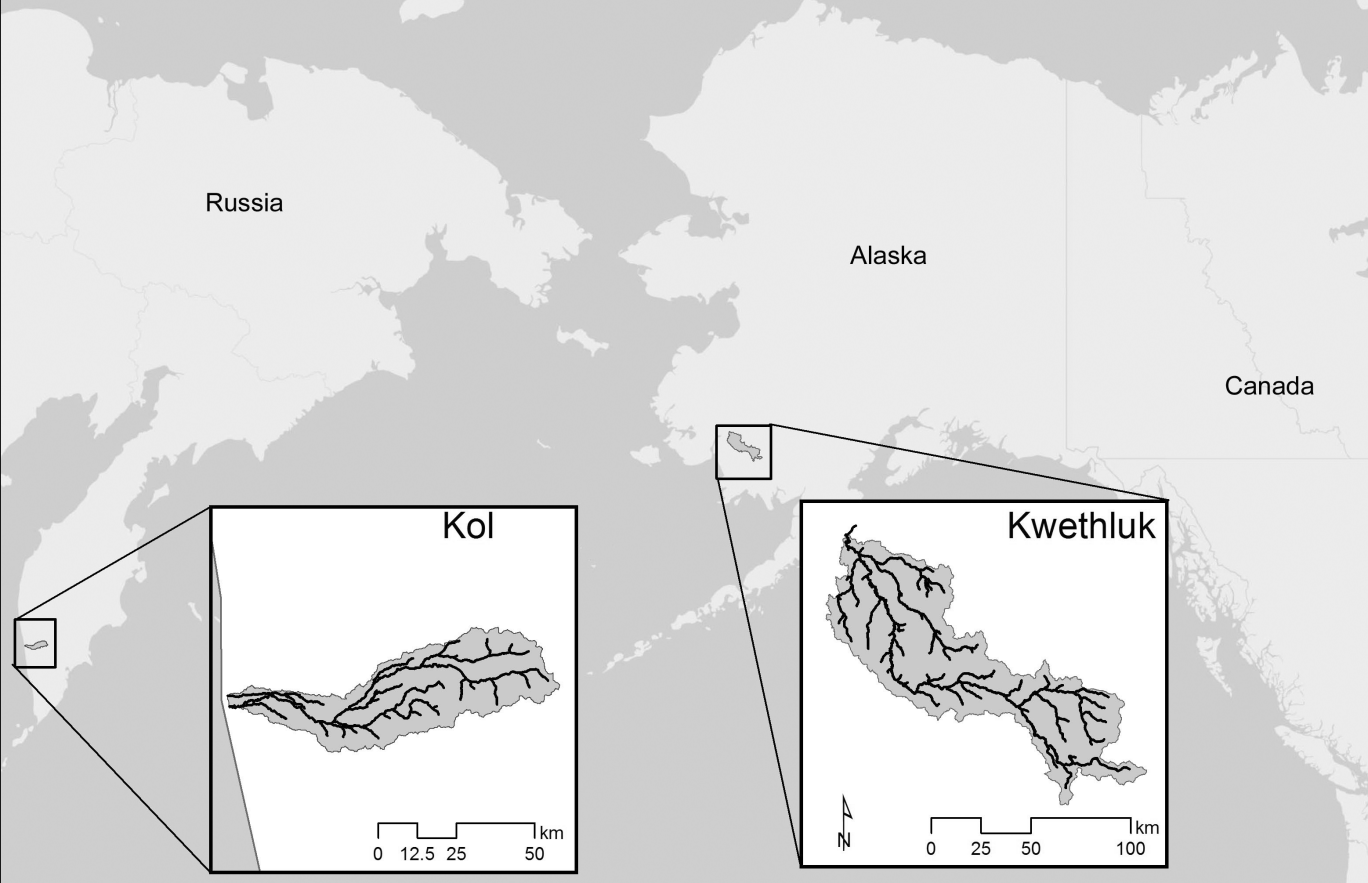
Locations of the two study rivers, the Kol River on the Kamchatka peninsula, Russian Federation, and the Kwethluk River, a tributary of the Kuskokwim in western Alaska.

**Figure 2:**
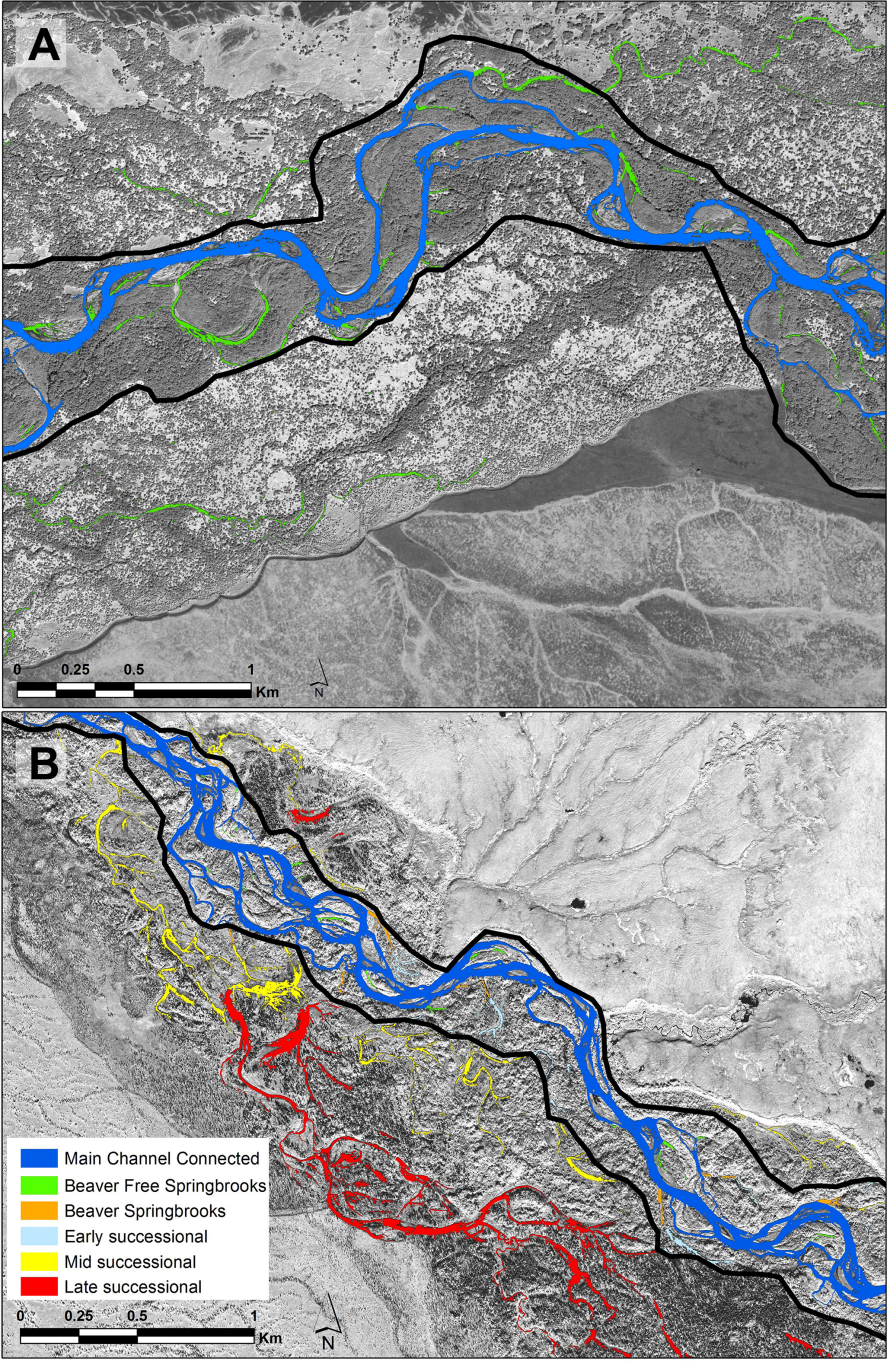
Portions of each of the study floodplains contrasting the Kol (A) without beavers and the Kwethluk (B) with 80 percent of the off-channel habitats dammed by beavers. Dark blue, main channel; Green, beaver-free springbrooks; Orange, beaver-influenced springbrooks; Light blue, early-successional ponds; Yellow, mid-successional ponds; and Red, Late-successional ponds.

## Materials & Methods

### Study areas

The Kol River (5th order) originates in the Central Mountain Range of the Kamchatka Peninsula, Russia (Fig. 1). Floodwaters commonly inundate the expansive floodplains to the valley walls in the spring and fall (Eberle & Stanford, 2010). The study reach is an expansive anastomosing section of the river where channel avulsions create a complex network of flood channels that disconnect from the main channel through sediment accretion or levees mediated by wood jams (Fig. 2). The Kol is an extremely productive salmon river with 3–10 million spawners returning annually (6 salmon species + anadromous *O. mykiss* and 2 char species). Pink salmon dominate anadromous runs with 5–7 million returning on even years and approximately 500,000 on odd years (Pavlov et al., 2009). This productivity is largely due to a huge marine derived nutrient (MDN) subsidy and extensive networks of springbrooks in both the parafluvial and orthofluvial zones which have strong trophic linkages to an expansive and productive floodplain forest (Eberle & Stanford, 2010; Morris & Stanford, 2011).

The Kwethluk River (5th order) is a tributary of the Kuskokwim River on the west coast of Alaska. Similar to the Kol, floodwaters inundate the floodplains in the spring and fall. The study reach is an expansive anastomosing floodplain located between 37 and 64 km above the confluence of the Kwethluk with the Kuskokwim (Fig. 1). A complex network of flood channels is present and springbrooks persist at base flow in abandoned flood channels (Fig. 2). However, due to abundant beaver damming of springbrooks combined with riparian plant succession over time, a mosaic of early-, mid-, and late-successional beaver ponds are distributed throughout the parafluvial and orthofluvial zones of the river (as described by Mouw et al., 2013). Dammed springbrooks form ponds that often have multiple dams and ponds in sequence or, alternatively, have one or two dams and ponds that feed a springbrook to the river confluence (beaver-influenced springbrook). In fact, 80% of all off-channel habitats are located behind beaver dams (Malison et al., 2014) and the main consequence of this extensive damming is that only early-successional ponds are heavily used by salmon; whereas, very few, or no salmon at all, are present in the abundant mid- to late-successional ponds. See Malison et al. (2014) for thorough descriptions of how beavers influence the quantities, distribution, and properties of each habitat type on the floodplain. The Kwethluk has far fewer fish than the Kol, but it is an important salmon river in the Kuskokwim basin, Alaska. Up to ∼200,000 salmon return annually (all 5 salmon species but *O.mykiss* is resident only—non-anadromous) with coho and chum (*O. keta*) dominating the runs. Thus, over a two year period, the total run size of all salmonids in the Kwethluk River averages about 3% of the total run size of all salmonids in the Kol River, resulting in a large difference in the amount of marine derived nutrients.

Based on habitat availability, both the Kol and Kwethluk Rivers have high complexity and high production potential for rearing juvenile salmon compared to other rivers located around the Pacific Rim (Luck et al., 2010; Whited et al., 2013). Both rivers also have a long legacy of commercial and subsistence salmon harvest; however, we estimate that long-term harvest rates likely are 2–3× higher in the Kwethluk because the Kol is largely uninhabited, whereas the Kwethluk Village sits at the confluence of the Kwethluk and the main Kuskokwim River, and town of Bethel is located downstream, both providing fishing pressure (Augerot, 2005; Pavlov et al., 2009).

### Data collection

#### Floodplain characteristics and rearing habitat availability

To compare floodplain characteristics between the two rivers, we obtained habitat complexity metrics from the Riverscape Analysis Project (a publicly available geospatial database of the physical structure of basins around the Pacific Rim, Whited et al., 2012). We quantified springbrooks, beaver ponds, and main channel shallow shorelines at base flow by classifying Quickbird satellite multispectral imagery collected on the Kol in 2004 and the Kwethluk in 2011 (1 m pixel resolution). Pixels associated with water bodies were classified and delineated using Definiens Developer (version 8.6; Definiens, Westminster, Colorado; www.ecognition.com) and Erdas Imagine (version 9.3; Intergraph, Norcross, GA; http://www.hexagongeospatial.com/products/producer-suite/erdas-imagine) software. Following delineation, habitat patches (i.e., ponds, springbrooks, and shallow shorelines) were demarcated by manually drawing polygons around features in Arc/Map (version 10; ESRI, Redlands, California, USA; www.esri.com), following similar methods used by Whited et al. (2013).

#### Juvenile salmon metrics

In the spring, summer, and fall, we sampled juvenile salmon in main channel shallow shoreline, tributary, and parafluvial and orthofluvial springbrook sites on the Kol (2004–2008) and Kwethluk River floodplains (2006–2008) through the Salmonid Rivers Observatory Network Program (SaRON), a research and conservation cooperative of the Flathead Lake Biological Station, Moscow State University, and the Wild Salmon Center (Portland, OR). Sampling sites within in each habitat type were distributed throughout the length of each floodplain. We determined fish density by species in lotic habitats using 3-pass depletion electrofishing over 50 m reaches delimited by block nets. We sampled juvenile salmon in beaver ponds (of all successional stages) in 2006 and 2009–2011 through the SaRON program (Malison et al., 2014). Ponds were sampled by depletion minnow trapping in 2006 and by using mark-recapture minnow trapping and PIT-tagging in 2009–2011. Fish were held in buckets with aerators, anesthetized with clove oil or MS-222, and then identified, measured, and weighed. We calculated fish population densities from data collected in both rivers from 2004–2008 using Bayesian inference for depletion estimates (Wyatt, 2002). We calculated Fulton’s condition factor, *K* (Ricker, 1975), for each fish by dividing mass (g) by length (mm) cubed and multiplying by a scalar of 10^5^ to determine if condition varied by habitat type or by river floodplain. We calculated % daily batch growth rates in mass (*m*_*n*_) for coho and Chinook in each habitat type in both rivers. Percent daily growth in mass was calculated using the formula: ((*m*_2_ − *m*_1_)∕(*m*_1∗_(*t*_2_ − *t*_1_)))∗100. To determine accuracy of batch growth rates we compared batch growth rates to individual growth rates (from PIT-tagged individuals). Research done on vertebrate animals was approved by the University of Montana Institutional Animal Care and Use Committee, protocol identification number 014-08JSDBS-040108. The State of Alaska Department of Fish and Game approved field permits (SF-2005-086, SF-2006-125, SF2007-105, SF2008-082, SF2009-102, SF2010-088, SF2011-066). Work on the Kol River was conducted in partnership with KamchatNIRO and the Russian Academy of Sciences and permission was granted to conduct field studies.

#### Floodplain production

We estimated the biomass produced from parafluvial and orthofluvial habitats in both floodplains by extrapolating biomass/m^2^ (calculated from batch growth rates and density estimates) to the total area of each habitat type for the time period between June and August. Because growth data were sparse for Chinook, we used coho growth rates for the production estimates. We used combined densities of coho and Chinook in production calculations. We calculated production from off-channel habitats for both river floodplains (i.e., parafluvial and orthofluvial springbrooks in the Kol and beaver-free springbrooks, beaver-influenced springbrooks, and early-, mid-, and late-successional beaver ponds in the Kwethluk). We also estimated the number of individuals that reared in each habitat type because it is possible that patterns in biomass accumulation may be different from the number of individuals reared.

We estimated what floodplain scale production of juvenile coho and Chinook would be in the Kwethluk if beavers were absent and early-successional beaver ponds were replaced by free flowing springbrooks in the parafluvial zone and if mid- and late-successional beaver ponds were replaced by free flowing springbrooks in the orthofluvial zone. We used satellite imagery in ArcMap to delineate channel courses (i.e., previous springbrook flowpaths) in beaver ponds and grossly calculated springbrook areas that would be present had they not been dammed by beavers. This was by necessity a rough calculation and we assumed that spring brooks would be present where there currently are beaver dams. It is very possible that there could be more or less area covered by spring brooks than we estimated if beavers were absent from the system. However, during our years of research on the Kwethluk, we observed construction of several new beaver dams on parafluvial spring brooks in recent avulsion channels and in every case, the dams were placed below upwelling zones which ensured pond volume at river base flows. In order to estimate potential floodplain production without beavers in the Kwethluk River we used density and growth estimates measured in parafluvial beaver-free springbrooks of the Kwethluk River and multiplied them by the total amount of springbrook habitat that would be present in the absence of beavers (i.e., the amount of springbrook habitat that would replace early-, mid-, and late-successional beaver ponds). We assumed that density and growth measurements would be similar in all springbrooks in both the parafluvial and orthofluvial zones of the Kwethluk River in the absence of beavers because density and growth were similar in all parafluvial and orthofluvial springbrooks in the Kol River (see results below).

Finally, we made a rough estimate of how floodplain production might change in the Kol River if beavers were introduced. To do this we assumed that orthofluvial spring brooks would be replaced by mid- and late-successional beaver ponds and that some parafluvial spring brooks would be converted to early-successional beaver ponds. We estimated the amount of each habitat type that would be present based on the proportion of each habitat type present on the Kwethluk River. We used density and growth data measured in each respective type of beaver pond in the Kwethluk River to estimate production if all three types of beaver ponds replaced similar proportions of springbrook habitat on the Kol River as is currently present on the Kwethluk River. We used growth rates from fish rearing in beaver ponds in the Kwethluk River to make these estimates for the Kol River because there are no beaver ponds in the Kol River. However, it is possible that if beaver ponds were present on the Kol River, that fish would grow even faster in them as compared fish in Kwethluk ponds because of the large subsidy of marine derived nutrients that the Kol River receives.

#### Statistical analyses

We analyzed the effect of habitat type on the number of species, density, condition, and batch growth rates of juvenile coho and Chinook in each river using one-way analysis of variance (ANOVA)(PROC GLM, SAS Institute, Cary, North Carolina). Pairwise comparisons among the least squares means for habitat type were assessed using Tukey’s honestly significant difference (HSD). All data sets were tested for normality and, where necessary, log10 transformed in SAS to meet assumptions of normality and homogeneity of variance prior to statistical analysis. If variance was not homogeneous, we used Welch’s ANOVA.

Comparisons of the effect of river type (with or without beavers) were made using *t*-tests. Because multiple comparisons were done for each variable (for multiple habitat type comparisons), a Bonferroni correction factor (Rice, 1989) was used to determine the significance level by dividing 0.05 by the number of tests. All statistical tests were considered significant where *P* < 0.05, unless a Bonferroni correction was applied.

## Results

### Comparative floodplain characteristics

The Kwethluk has a larger catchment area and total floodplain area than the Kol, but the mean floodplain elevation, number of floodplains, floodplain to watershed ratio, floodplain sinuosity, and number of nodes are similar for both rivers (Table 1). The Kol has over twice as many nodes (channel separations and confluences) per floodplain length as the Kwethluk (i.e., the channel network of the Kol floodplain is more complex than the Kwethluk). The focal floodplains have similar slopes (Kwethluk, 0.0020 and Kol, 0.0022), width (Kwethluk, 42 m and Kol, 50 m) and types of sediment supplies (dominated by gravel and cobble) indicating that the primary physical drivers of floodplain complexity are similar. Within the focal flood plains, the Kol River has 1.5× more total aquatic habitat than the Kwethluk but the percentage of off-channel habitat is similar for both rivers (20.5 vs. 22.6%, Table 1). However, over 99% of off-channel habitats in the Kol are comprised of parafluvial (55%) and orthofluvial (45%) spring brooks. While in the Kwethluk, only 17% of the off-channel habitat is comprised of spring brooks because the majority (80%) of the off-channel habitat is composed of spring brooks that have been converted to beaver ponds of varying successional stages (described by Mouw et al., 2013).

**Table 1:** Watershed and focal floodplain characteristics for the Kwethluk River, Alaska and the Kol River, Kamchatka, Russian Federation.

	Kwethluk	Kol
Watershed area	3,846 km^2^	1,502 km^2^
Total floodplain area	2.49 × 10^8^ m^2^	1.04 × 10^8^ m^2^
Mean floodplain elevation	212 m	280 m
Floodplains (#)	10	8
Floodplain: watershed ratio	0.06	0.07
Floodplain sinuosity	1.68	1.20
Nodes (#)	224	192
Nodes per length of floodplain	0.86	1.81
Total aquatic habitat	283 ha	409 ha
Main channel total area	219 ha	325 ha
Off-channel habitat area	64 ha	84 ha
Springbrook total area	11 ha	83 ha
% off-channel springbrook area	0.17	0.99
Beaver pond area	51 ha	0
% off-channel beaver pond area	0.80	0

### Species composition

Within the Kol River, all twelve fish species known to occur in Kamchatka were found in all habitat types, including rainbow trout (*Oncorhynchus mykiss*), coho (*O. kisutch*), Chinook (*O. tshawytscha*), chum (*O. keta*) and sockeye salmon (*O. nerka*), cherry salmon (*O. masou*), pink salmon (*O. gorbushcha*), Dolly Varden char (*Salvelinus malma*), white spotted char (*S. leucomaenis*), three-spined stickleback (*Gasterosteus aculeatus*), and ninespine stickleback (*Pungitius pungitius*), plus lamprey (*Lethenteron camschaticum*). Coho were most abundant in orthofluvial springbrooks and least abundant in main channel habitats. The number of total fish and salmon species was similar for all Kol floodplain habitats (*F*_[3,22]_ = 1.72, *P* = 0.1919 and *F*_[3,22]_ = 1.57, *P* = 0.2251).

Within the Kwethluk floodplain, coho and Chinook salmon were codominant in backwaters, and coho salmon dominated springbrooks and beaver ponds. In contrast to the Kol, the total number of fish species varied significantly with habitat type in the Kwethluk (*F*_[8,53]_ = 3.03, *P* = 0.0070). Significantly more species were in parafluvial springbrooks and early-successional beaver ponds than in late-successional ponds (Tukey’s HSD, *P* < 0.0361). The number of salmon species also varied by habitat type (*F*_[8,15.94]_ = 2.50, *P*0.0566), with 1.7–2× less species of salmon present in late-successional ponds compared to all other habitats (Tukey’s HSD, *P* < 0.0476). Other species also present include rainbow trout, chum salmon, sockeye salmon, Dolly Varden, three-spine stickleback, lamprey, arctic grayling (*Thymallus arcticus*), slimy sculpin (*Cottus cognatus*), round whitefish (*Prosopium cylindraceum*), and Alaska blackfish (*Dallia pectoralis*).

Though a large run of pink salmon returns to the Kol River, coho predominated in off-channel habitats in both rivers (Kol: 44% and Kwethluk: 46%). Chinook salmon were less abundant in off channel habitats of the Kol vs. the Kwethluk (3 vs. 25%). Coho, sockeye (*Oncorhynchus nerka*), and lamprey (*Lethenteron camschaticum*) made up greater proportions of the fish in orthofluvial vs. parafluvial habitats in the Kol River.

### Density

**Figure 3:**
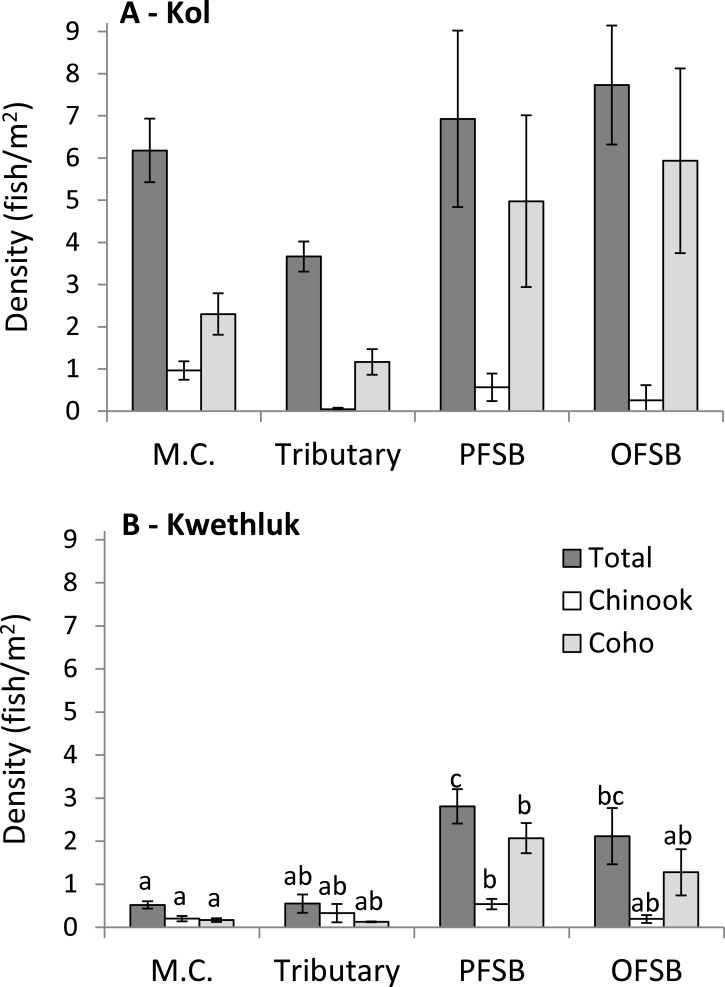
Density (± 1 SE) of all salmonids (totals) and Chinook and coho in main channel (M.C.), tributary, parafluvial springbrook (PFSB), and orthofluvial springbrook habitats (OFSB) for the Kol (A) and the Kwethluk (B) Rivers. T-tests contrasting habitat types were significant at *p* < 0.05.

In the Kol River, densities of total fish, coho, and Chinook were similar in all habitats (*F*_[3,28]_ = 2.70, *P* = 0.065, *F*_[3,7.7543]_ = 3.06, *P* = 0.0937, and *F*_[3,5.6905]_ = 3.15, *P* = 0.1125; Fig. 3A).

In contrast, densities of all fish, coho, and Chinook varied by habitat type in the Kwethluk River (*F*_[3,28]_ = 12.8, *P* < 0.0001, *F*_[3,28]_ = 6.37, *P* = 0.002, and *F*_[3,28]_ = 2.94, *P* = 0.0505; Fig. 3B). Main channel habitats had lower total densities than off-channel parafluvial and orthofluvial springbrooks (Tukey’s HSD, *P* < 0.0099). Tributaries had lower total densities than parafluvial springbrooks (Tukey’s HSD, *P* = 0.0069). Coho and Chinook densities were lower in main channel habitats vs. parafluvial springbrooks (Tukey’s HSD, *P* = 0.0014 and *P* = 0.0462). Densities were 3–12× lower in mid- and late-successional beaver ponds than in main channel, tributary, and springbrooks habitats (Malison et al., 2014).

All habitats of the Kol had higher total densities compared to the Kwethluk (*t* > 2.18, *P* < 0.0112), except for parafluvial springbrooks (*t* = 2.57, *P* = 0.1108). Coho densities were higher in main channel habitats in the Kol vs. the Kwethluk (*t* = 2.20, *P* = 0.0012), but similar for other habitats. Chinook densities were higher in Kol vs. Kwethluk main channel habitats (*t* = 2.16, *P* = 0.0052).

### Fish condition and growth

#### Fish condition

Coho in main channel, parafluvial, and orthofluvial springbrook habitats all had similar condition factors in the Kol River (*F*_[2,15]_ = 0.82, *P* = 0.4582). However, the condition of Chinook varied by habitat type (*F*_[2,12]_ = 7.49, *P* = 0.0077), being higher in orthofluvial vs. parafluvial springbrooks (Tukey’s HSD, *P* = 0.0107) and higher in main channel habitats vs. parafluvial springbrooks (Tukey’s HSD, *P* = 0.0317).

In the Kwethluk, the condition of coho varied significantly by habitat type (*F*_[6,15.4581]_ = 10.28, *P* = 0.0001). Main channel coho had lower conditions than coho in all other habitat types (Tukey’s HSD, *P* < 0.0015), except for orthofluvial springbrooks, which were similar to the main channel. The condition of Chinook did not differ by habitat type (*F*_[6,39]_ = 1.45, *P* = 0.2204).

Comparing the two rivers, we found that coho were in better condition in all habitat types in the Kol vs. the Kwethluk (*t* > 2.11, *P* < 0.0043) in spite of much higher densities in the Kol. Chinook were in better condition in main channel and orthofluvial habitats in the Kol vs. the Kwethluk (*t* > 2.13, *P* < 0.0042).

#### Growth rates

**Figure 4:**
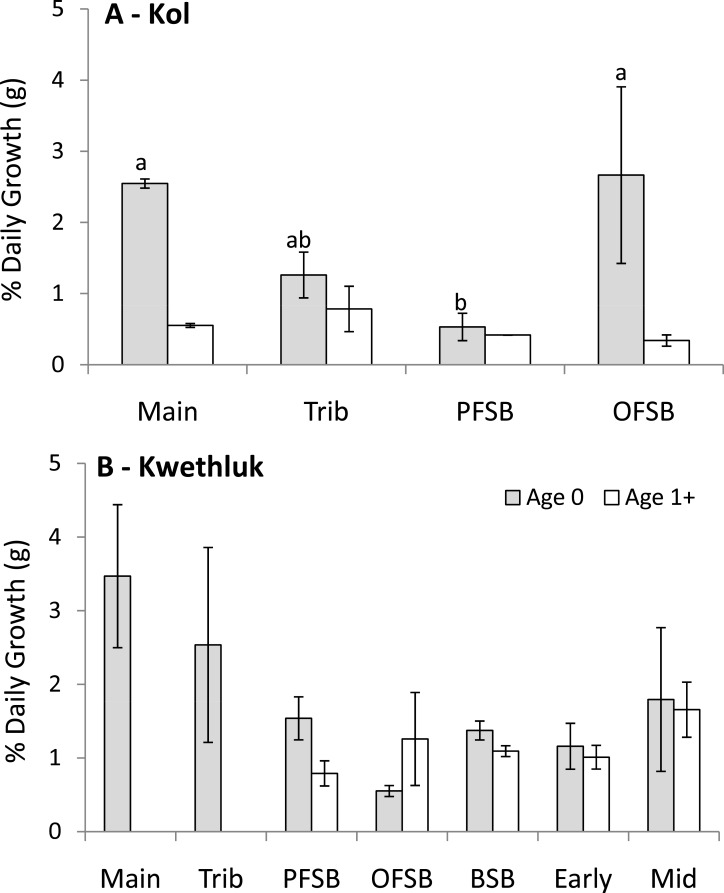
Mean (±1 SE) batch growth rates (mass per day) from June–August of unmarked coho sampled in habitats on (A) the Kol and (B) Kwethluk rivers (main, main channel shallow shorelines; Trib, tributaries; PFSB, parafluvial springbrooks; OFSB, orthofluvial springbrooks; BSB, beaver-influenced springbrooks; Early, Early-successional beaver ponds; and Mid, Mid-successional beaver ponds).

Within the Kol River, mean batch growth rates varied by habitat type for age-0 coho (*F*_[3,4]_ = 10.47, *P* = 0.023; Fig. 4A), with main channel and orthofluvial springbrook habitats having higher growth rates than parafluvial springbrooks (Tukey’s HSD, *P* < 0.029). Growth rates of age 1+ coho did not differ by habitat type (*F*_[3,5]_ = 2.28, *P* = 0.197).

Within the Kwethluk, mean batch growth rates were similar among all habitat types for age-0 and age 1+ coho (*F*_[6,18]_ = 1.64, *P* = 0.193 and *F*_[4,14]_ = 1.70, *P* = 0.207; Fig. 4B). In the one late-successional pond with juvenile salmon (other ponds had no salmon), batch growth rates were 2× lower than in springbrooks and 3–4× lower than in early- and mid-successional ponds.

In comparing the two rivers, age-0 coho grew almost 3× faster in parafluvial springbrooks in the Kwethluk than in the Kol, though the difference was not significant after Bonferroni correction (*t* = 2.36, *P* = 0.0166). Age-0 coho in main channel shallow shorelines, tributaries, and orthofluvial springbrooks grew at similar rates in both rivers (*P* > 0.2515). Age 1+ coho grew at similar rates in parafluvial and orthofluvial springbrooks in both rivers (*P* > 0.1375). Chinook also grew at similar rates in main channel habitats of both rivers (*t* = 4.30, *P* = 0.4684).

Batch growth rates differed significantly and underestimated growth compared to growth rates from individually tagged coho for age-0 fish (*F*_[4,45]_ = 20.64, *P* = < 0.0001). In contrast, growth rates calculated from individuals were similar to batch growth rates for age-1+ coho (*F*_[4,50]_ = 1.54, *P* = 0.2059). Using batch growth rates likely underestimates growth, mainly because newly emerged age-0 fish continually enter the sampling pool as they grow large enough to be captured. Though batch rates likely underestimate production estimates, we used the same methods in both floodplain reaches, allowing comparisons to be made.

### Floodplain scale production

#### The Kol River—without and with beavers

**Figure 5:**
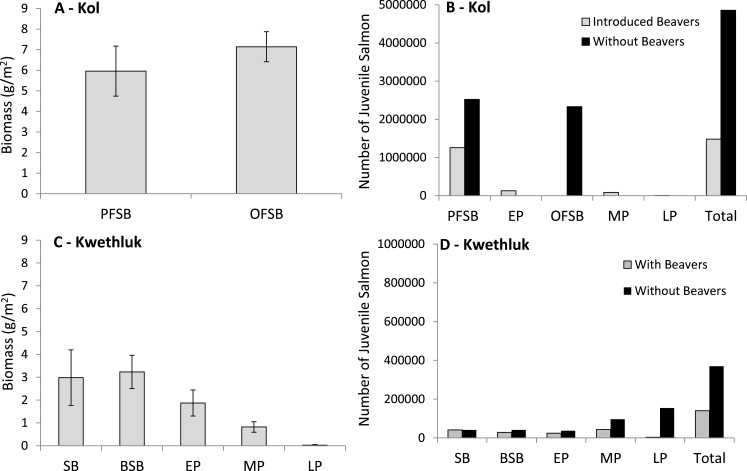
Mean (±1 SE) juvenile salmon biomass produced per unit area for off-channel floodplain habitats in (A) the Kol and (C) the Kwethluk and the total number of juvenile coho and Chinook produced by off-channel habitat type for the (B) Kol and (D) Kwethluk. Note the scale for the Kol (B) is five times larger than that of the Kwethluk (D) (PFSB, parafluvial springbrooks; OFSB, orthofluvial springbrooks; SB, beaver-free springbrooks; BSB, beaver-influenced springbrooks; EP, early-successional beaver ponds; MP, mid-successional beaver ponds; and LP, late-successional beaver ponds).

Parafluvial and orthofluvial springbrooks produced a similar biomass of juvenile coho and Chinook per square meter (Fig. 5A). The floodplain contained 45.5 ha of parafluvial springbrook habitat and 37.7 ha of orthorfluvial springbrook habitat. For the entire floodplain, we estimated that orthofluvial springbrooks produced 45 kg/day (2,692 kg in total) and reared 2,333,549 individuals while parafluvial springbrooks produced 45 kg/day (2,712 kg in total) and reared 2,523,407 individuals for a total of 5,404 kg and 4,856,956 individuals (Fig. 5B).

We estimated that orthofluvial springbrooks would be replaced by 48.7 ha of mid-successional beaver ponds and 35.1 ha of late-successional ponds (ponds that replace springbrooks cover a larger area). We estimated that in the orthofluvial zone mid-successional ponds would produce 6.7 kg/day (400 kg in total) and rear 87,096 individuals and that late-successional ponds would produce 0.1 kg/day (4 kg in total) and rear 6,293 individuals (Fig. 5B). In the parafluvial zone approximately half the parafluvial springbrooks would be replaced by 50.7 ha of early-successional beaver ponds (inundated ponds cover more area than springbrooks) and 22.7 ha of springbrook habitat would remain. We estimated that in the parafluvial zone parafluvial springbrooks would produce 22.5 kg/day (1,352 kg in total) and rear 1,258,321individuals and that early-successional ponds would produce 15.8 kg/day (949 kg in total) and rear 130,636 individuals (Fig. 5B). In total, if beavers were present on the floodplain, 2,705 kg (45 kg/day) would be produced and 1,482,346 individuals could rear (Fig. 5B). If beavers modified the Kol River as habitat has been modified on the Kwethluk River, we estimate that biomass production would decline by half and that three times fewer individuals would rear on the floodplain. This is largely due to the loss of juvenile salmon production from orthofluvial springbrooks that would be blocked by beaver dams and replaced with mid- and late-successional beaver ponds.

#### The Kwethluk River—with and without beavers

Biomass produced per square meter varied by habitat type on the Kwethluk (*F*_[4,14]_ = 4.61, *P* = 0.0139; Fig. 5C). The floodplain was comprised of 4.7 ha of beaver-free parafluvial springbrooks, 4.6 ha of parafluvial beaver-influenced springbrooks, 9.4 ha of early successional beaver ponds, 24.2 ha of mid-successional beaver ponds, and 17.5 ha of late successional beaver ponds. Production from the parafluvial zone (including beaver-free and beaver-influenced springbrooks, and early-successional beaver ponds) was estimated to be 7.7 kg/day (464 kg in total, <20% of the biomass produced in the same habitat in the Kol River) and 93,752 individuals were reared. We estimated that an additional 3.3 kg/day (199 kg in total) was produced and 43,354 individuals were reared in mid-successional beaver pond habitats on the Kwethluk (Fig. 5D). Production from late-successional ponds was extremely low, with almost 50× less salmon being produced compared to mid-successional ponds at 0.07 kg/day (4 kg in total), and only 3,132 individuals were reared (Fig. 5D). Combining all parafluvial and orthofluvial off-channel habitats, we estimate that 667 kg of biomass was produced and 140,238 juvenile coho were reared in the presence of beavers (Fig. 5D). Compared to the Kwethluk, the Kol River produces an order of magnitude greater biomass (in just two months) and rears almost forty times more juveniles per year from off-channel habitats.

Based on habitat availability in the Kol River, we assumed that if beavers were not present on the Kwethluk River, all off-channel habitats would be classified as free-flowing springbrooks connected to the main channel network. Assuming all other factors remained the same, besides replacing dammed ponds with springbrooks, we estimate that if early-successional ponds were replaced with parafluvial springbrooks, slightly less biomass would be produced (125 vs. 175 kg; 2.1 vs. 2.9 kg/day), but that 1.5× the individuals could be reared (37,142 vs. 24,072). If mid-successional ponds were replaced with orthofluvial springbrooks, 1.5× more biomass could be produced (287 vs. 199 kg; 4.8 vs. 3.3 kg/day) along with a two-fold increase in the number of individuals reared (96,335 vs. 43,354, Fig. 5D). Replacing late-successional ponds with orthofluvial springbrooks could result in a fifty-fold increase in production (207 vs. 4 kg; 3.4 vs. 0.1 kg/day, and 154,080 vs. 3,132 ind.; Fig. 5D). We estimated production of biomass could be 2× higher between June–August (1,174 vs. 667 kg), and almost 3× the number of individuals could be reared (369,713 vs. 140,238 ind.; Fig. 5D) if beavers were not present in the entire floodplain.

## Discussion

Within large alluvial river floodplains, beavers may reduce the potential production of juvenile salmon by blocking large portions of the floodplain from being used as rearing habitat. Though other factors, such as the size of adult salmon runs and the availability of marine derived nutrients, may be the primary drivers of differences in overall productivity between the Kwethluk and Kol Rivers, our data show that the loss of habitat for juvenile salmon in a beaver dominated floodplain could reduce salmon production. Springbrooks provide important rearing habitat for juvenile salmon but are also ideal locations for beaver dams. When significant numbers of springbrooks are converted into beaver ponds the usable portion of the floodplain shrinks, with a large proportion of rearing habitat located behind numerous beaver dams that are apparently impassable to juvenile salmonids. In the Kwethluk River 80% of the off channel habitat is comprised of beaver ponds and very few salmon rear in ponds within the orthofluvial zone. In comparison, juvenile salmonids were very abundant in springbrooks throughout the entire floodplain in both the parafluvial and orthofluvial zones in the Kol River, where there are no beavers and all springbrook habitats are interconnected with the main channel. In the Kwethluk River, low juvenile salmon densities in orthofluvial habitats were likely a result of limited access past extensive beaver dam complexes. It is possible that orthofluvial habitats were accessible, but not desirable (i.e., hypoxic), however Malison (2013) found that juvenile salmon placed in enclosures survived and grew in orthofluvial ponds in the summer months. If beaver dams did not block springbrooks in the orthofluvial zone, and all other factors remained the same, we infer that salmon production might be higher in the Kwethluk River than the current condition. However, other factors likely are limiting overall production in the Kwethluk relative to the Kol, as discussed below. Furthermore, if beavers were introduced to the Kol River and they built enough dams such that significant amounts of orthofluvial springbrooks were lost as rearing habitats, we expect that salmon production could decline dramatically. We expect that in other similar rivers systems, excessively high numbers of beaver dams may reduce juvenile salmon production by reducing connectivity of off channel habitats.

If insufficient access to springbrook rearing habitat due to blockage by beaver dams is limiting production, we estimate that less than half of the biomass and a third as many juvenile salmon are being reared on the Kwethluk floodplain as would be produced if beavers were absent. We assumed that parafluvial springbrooks would be present in place of early-successional beaver ponds and that orthofluvial springbrooks would be present in place of mid- and late-successional beaver ponds. However, it is possible that not all springbrooks would still be present and flowing if beavers were absent from the landscape. Beaver dams act to recharge and elevate alluvial groundwater levels throughout the floodplain. Thus, removal of beavers could result in dewatering of some springbrooks. Also, channels farthest from the main river in the depositional zone may be expected to fill with organic debris and fine sediments over time. However, some springbrooks would still exist in the orthofluvial zone if beavers were absent (i.e., they are present, interconnected, and heavily used by salmon in the Kol River) but it is difficult to know exactly how much of the habitat would be present. The magnitude of the increase in production would decline with increased dewatering and it is possible that our beaver-free production estimates could be biased high.

Although beaver ponds were unproductive in the orthofluvial zone, the opposite is true for the parafluvial zone of the Kwethluk river. Malison, Eby & Stanford (2015) found that the presence of early-successional beaver ponds in the parafluvial zone had a positive impact on salmon production. Ponds had lower salmon densities and higher growth rates, and because ponds covered a greater area than either type of springbrook, more salmon biomass was produced in the parafluvial zone. This results in a tradeoff with positive impacts for salmon rearing in beaver ponds in the parafluvial zone where frequent inundation provides pathways for salmon to move in and out of ponds, vs. a negative impact for salmon because they cannot use habitat in the orthofluvial zone. The overall impact of beavers in the Kwethluk could be negative because of the extensive dam complexes and large amount of habitat lost in the orthofluvial zone, relative to the small amount of beneficial beaver habitat in the parafluvial zone. However, if the degree of habitat modification was not so high it is possible that the positive effects of beavers would outweigh the apparent negative effects.

It is possible that there could be an optimal density of beavers in river floodplains where the presence of beavers increases habitat variation and the range of growth opportunities for juvenile salmon (Malison, Eby & Stanford, 2015), but there are not so many beavers that entire portions of the floodplain are blocked from use. However, it is difficult to determine how different numbers of beavers will influence a large river floodplain over time. In recent decades, there has been a decrease in trapping pressure, which has allowed beaver populations to expand in Alaska. It is not clear how many beavers would have to be present in the floodplain over time to create the current degree of habitat modification in the Kwethluk River. Dams built in the orthofluvial zone last a long time because they are not subjected to scouring flows, except possibly in extreme flow events, and it is not clear what flood levels would be required to inundate and/or scour these habitats. Beavers also have the ability to spread rapidly in systems where they are free from predation, as has been shown by the Tierra del Fuego introduction (Lizarralde, 1993). Intact large river floodplains have a plethora of ideal beaver habitat (i.e., low gradient, low flow, upwelling water, abundant food resources) providing ample opportunity for population expansion. Approximately half of the juvenile salmon produced in the Kol river rear in orthofluvial springbrooks. If beavers were introduced and built dam complexes on these springbrooks this could have a large negative impact on salmon production. Thus, we conclude that significant beaver modification of springbrook habitats could substantially reduce production potential of the Kol River. Other large alluvial rivers with expansive floodplains could be similarly affected.

We estimated that production from the Kol River was an order of magnitude higher in biomass and that 40× more individuals could be reared compared to the Kwethluk with beavers. However, factors other than available rearing habitat and the presence of beavers, such as spawner numbers and river basin fertility likely strongly contribute to higher production in the Kol River. We know that reductions in escapement (i.e., number of returning spawners) can strongly impact food availability and growth rates of juvenile salmon by influencing riverine fertility (Bilby, Fransen & Bisson, 1996; Wipfli et al., 2003). The spawning and death of salmon clearly influences the overall productivity of streams (Richey, 1975; Wipfli, Hudson & Caouette, 1998; Morris & Stanford, 2011) and this subsidy of marine derived nutrients (MDN) induces positive feedback loops where increases in juvenile salmon production occurs in response to increased MDN in productivity of multiple trophic levels (Wipfli, Hudson & Caouette, 1998; Schindler et al., 2005). Thus differences in escapement between the Kwethluk and Kol should result in differences in fertility. Indeed, Morris & Stanford (2011) found that rivers around the Pacific Rim varied with relation to MDN loading from returning salmon, and that the Kwethluk exhibited much lower foliar *δ*^15^N than the Kol. Thus, the Kwethluk likely has a an overall lower production potential because of reduced escapement and fertility owing to high harvest rates by commercial and subsistence fishers. Nonetheless, it is quite clear that beavers reduce the availability of rearing habitat for salmon on the Kwethluk floodplain compared to the Kol.

However, if beavers were substantially reduced on the Kwethluk, salmon production might not change much owing to legacy effects on floodplain habitat and strong interactions with salmon harvest. Additionally, rearing habitat would have to be the limiting factor for salmon production to increase after removing beavers from the orthofluvial zone and it isn’t likely that this is the case at recent escapement levels. Indeed, coho and Chinook escapement has declined by 12–15× over the past nine years (Miller & Harper, 2012), probably owing to high commercial and subsistence harvest rates although density dependent limitation of production cannot be discounted without careful analysis of stock-recruitment relationships. In any case, we documented much higher densities of juvenile salmon in good condition in the Kol River than the Kwethluk River (up to 14.9 coho/m^2^). The Kol floodplain has a much higher capacity to produce salmon smolts than the Kwethluk River. Even if beavers were not present on the Kwethluk and the increased production potential was fully utilized, we show that the Kol River could produce 5 times the biomass and 14 times the individuals as the Kwethluk might without beavers, likely due to the 1–2 orders of magnitude higher salmon escapement levels that the Kol receives. Regardless of the potential for beavers to influence salmon production as shown herein, beavers and salmon have been part of North American riverscapes since at least the early Pliocene (Tedford & Harington, 2003). Beavers are strong interactors that naturally influence river ecology (Pollock, Beechie & Jordan, 2007; Burchsted et al., 2010; Mouw et al., 2013; Malison et al., 2014) and should be viewed in that context.

## Conclusions

The potential impact of beavers on larger salmon rivers should be considered in light of widespread reintroduction activities. Because the effects of beavers on stream fishes are so variable and site dependent (Kemp et al., 2012), each potential case for restoration should be examined critically. There is strong potential for beaver reintroductions to be beneficial for salmonids in small incised streams (Pollock et al., 2012) and perhaps as a way to mediate effects of climate change (Beechie et al., 2013), but reintroduction efforts are likely not beneficial in all cases. We expect that the presence of beavers on the productive rivers of the Kamchatka peninsula would be very detrimental for salmon populations, because beavers would likely block off-channel habitats that are currently important nursery habitats.

##  Supplemental Information

10.7717/peerj.2403/supp-1 Supplemental Information 1 Raw Data Set 1

10.7717/peerj.2403/supp-2 Supplemental Information 2 Raw Data Set 2

10.7717/peerj.2403/supp-3 Supplemental Information 3 Raw Data Set 3

## Additional Information and Declarations

### Field Study Permissions

The following information was supplied relating to field study approvals (i.e., approving body and any reference numbers): State of Alaska Department of Fish and Game. Permit #’s: SF-2005-086, SF-2006-125, SF2007-105, SF2008-082, SF2009-102, SF2010-088, SF2011-066. Work on the Kol River was conducted in collaboration with KamchatNIRO and the Russian Academy of Science. Permission was granted to conduct field studies.

### Data Availability

The following information was supplied regarding data availability: The raw data was supplied as Supplemental Information.
